# Inactivated autograft–prosthesis composite have a role for grade III giant cell tumor of bone around the knee

**DOI:** 10.1186/1471-2474-14-319

**Published:** 2013-11-09

**Authors:** SongFeng Xu, XiuChun Yu, Ming Xu, ZhiHou Fu

**Affiliations:** 1 Department of Orthopaedics, General Hospital of Ji’Nan Military Region, Ji’Nan 250031, China

**Keywords:** Giant cell tumor of bone (GCT), Autograft prosthesis composite, Knee

## Abstract

**Background:**

Giant cell tumors (GCT) around the knee are common and pose a special problem of reconstruction after tumor excision, especially for grade III GCT. We questioned whether en bloc resection and reconstruction with alcohol inactivated autograft-prosthesis composite would provide (1) local control and long-term survival and (2) useful limb function in patients who had grade III GCT around the knee.

**Methods:**

We retrospectively reviewed eight patients (5 males and 3 females) treated with this procedure with mean age of 31 years (range 20 to 43 years) from Jan 2007 to Oct 2008. 5 lesions were located in distal femur and 3 in proximal tibia. 4 patients were with primary tumor and the other 4 with recurrence. 2 patients showed pathological fracture.

**Results:**

Mean Follow-up is 54 months ranging from 38 to 47 months. No recurrence, metastasis, prosthesis loosening were found. The mean healing time between autograft and host bone was 5.5 months. The mean MSTS score was 26.3 (88%) ranging from 25 to 29. The mean ISOLS composite graft score was 32.8 (88.5%) ranging from 28 to 35. Creeping substitution is possibly the main way in bony junction. The healing time in femoral lesion is faster than that in tibial lesion.

**Conclusions:**

The technique of alcohol inactivated autograft-prosthesis composite could be able to achieve satisfactory oncological and functional outcomes in Grade III GCT.

## Background

Giant cell tumor (GCT) of bone is aggressive, potentially malignant lesion which remains a difficult and challenging management problem. The ends of long bones in skeletally mature individuals are involved in more than 80% of cases and 75% of them occur around the knee joint [1]. The reconstruction of large bone defects and restoration of joint function resulting from resection of aggressive grade III GCT around knee remains a challenging problem.

Since the local behavior of giant cell tumors can be aggressive and they have a greater risk of local recurrence, some authors advocate *en bloc* resection and reconstruction for these grade III lesions from the point of view of preventing local recurrence rate and preserving joint function [2,3]. Three most popular reconstructive methods are endoprosthesis, allograft–prosthesis composite and biological reconstruction. However, each of these methods has its short- and long-term advantages and disadvantages [4]. We noted that mega-prosthesis is a feasible method for treating GCT of grade III around the knee, and complications related to which were mainly prosthesis loosening and limb shortening, increase gradually with longer survival time [5].

We questioned whether en bloc resection and reconstruction with alcohol inactivated autograft-prosthesis composite would provide (1) local control and long-term survival and (2) useful limb function in patients who had grade III GCT around the knee. To overcome possible disadvantages of the above methods, 8 patients with grade III GCT around knee were treated with this method. The objectives were to evaluate whether this method can provide both mechanical stability in early time and fine limb function in long time on the basis of complete resection and decreasing recurrence rate.

## Methods

Collection of retrospective clinical data and the publication of the data were in accordance with local guidelines for research ethics and were approved by General Hospital of Ji'Nan Military Region. All patients had been treated the procedure by the same chief surgeon (XCY) and his assistants (SFX and MX) in the same institute. All procedures were in compliance with the Helsinki Declaration.

We retrospectively reviewed 8 patients with aggressive GCT around knee who underwent reconstruction using an autograft–prosthesis composite from Jan 2007 to Oct 2008 (Table 1). There were 5 males and 3 females with an average age of 31 years (range 20–43 years). 5 lesions located in distal femur and 3 in proximal tibia. 4 patients were with primary tumor and the other 4 with recurrence. 2 patients were with pathological fracture. All patients were diagnosed as GCT of Campanacci III determined by biopsy and pathology.

**Table 1:** Details of grade III GCT patients treated with autograft-prosthesis composite

**Site**	**Pathological fracture**	**Status of tumor**	**Follow-up (months)**	**MSTS function score**	**ISOLS image score**	**Status at latest follow-up**
Left distal femur	No	Recurrence	67	27 (90%)	33 (92%)	Disease-free
Right distal femur	No	Recurrence	60	26 (87%)	35 (97%)	Disease-free
Left distal femur	Yes	Primary	51	25 (83%)	28 (78%)	Disease-free
Left distal femur	No	Recurrence	48	25 (83%)	31 (86%)	Disease-free
Left distal femur	No	Primary	44	26 (87%)	30 (83%)	Disease-free
Right proximal tibia	No	Recurrence	38	29 (97%)	35 (97%)	Disease-free
Left proximal tibia	No	Primary	48	26 (87%)	30 (83%)	Disease-free
Right proximal tibia	No	Primary	48	27 (90%)	33 (92%)	Disease-free

The indications for a wide resection were extensive tumor (Campanacci III [6]) invasion with or without pathological fracture and rapid or large recurrent tumor after intensive curettage. In addition, according to Yang’s study [7], if the diameter of GCT lesion around the knee is over 1/2 on CT transversal image, *en bloc* resection and reconstruction should be chosen. All patients were in line with the above criteria.

**Figure 1:**
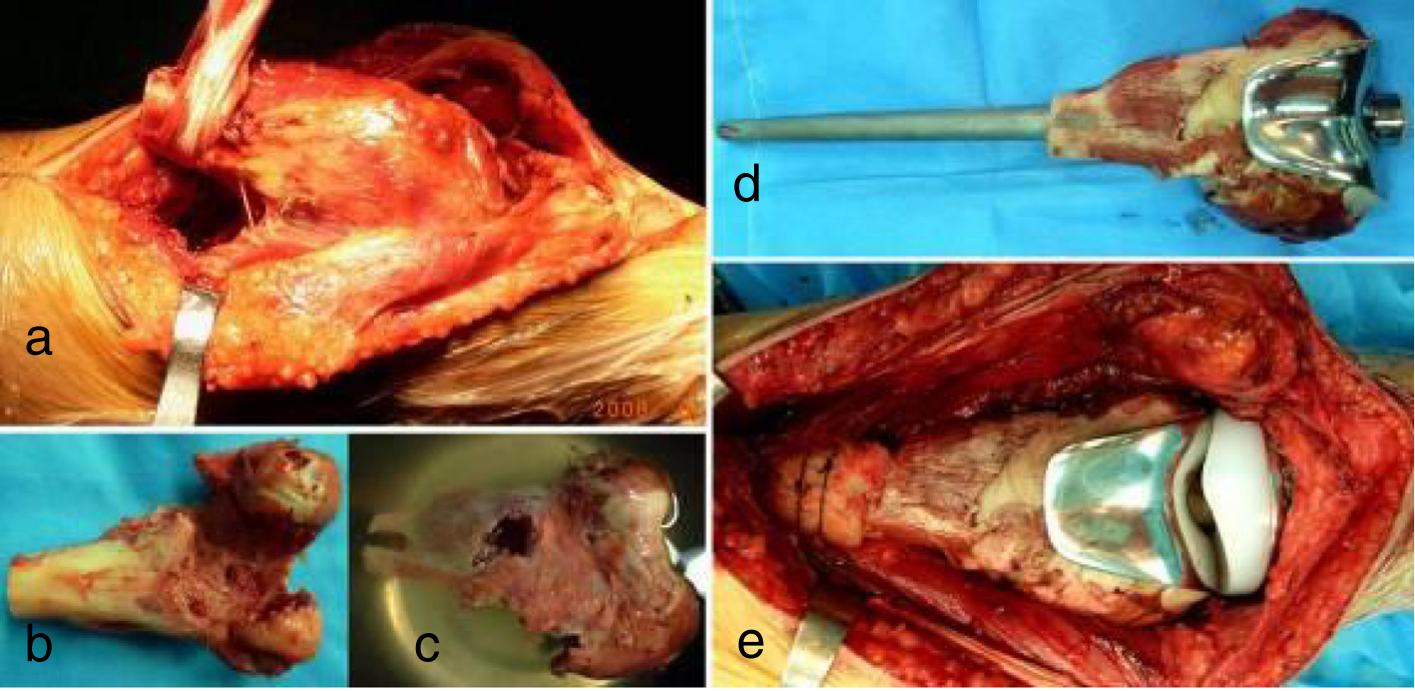
**A patient with postoperative recurrence of bone giant cell tumor and pathological fracture in left distal femur (Case 4). a** Osteotomy was performed at 3 cm above the upper boder of tumor, **b** All tissue on the tumor bone was removed and the medullary cavity was drilled through, **c** The tumor bone was infused in dehydrated alcohol for 30 minutes, **d** The deactivated autograft was adjusted and combined with prothesis using bone cement with which the bone defect filled **e** The autograft-prothesis composite was fixed with host bone with the junction site tied around autogenous bone as extracortical bone grafting.

All patients underwent *en bloc* resection of tumor and reconstruction with alcohol inactivated autograft–prosthesis composite under epidural anesthesia. The domestic rotating-hinged knee prosthesis (Lidakang, Beijing, China) were chosen. The conventional anterormedial incision encircling the biopsy scar for the knee was used. A length 3 cm longer than the tumor boundary was generally accepted. The surgical technique, taking the inactivated autograft-prosthesis composite of distal femur as a sample, was described as follows: (1) The lesion in distal femur was resected according to tumor-free technique rules, and then soft tissue and extraosseous tumor were cleared off (Figure 1a). (2) The medullar cavity was reamed and intraosseous tumor was curetted with the distal femoral articular surface removed (Figure 1b). (3) Preliminary screw fixation was prepared with cemented technique. The prepared autograft was then immerged into 99% alcohol for 30 minutes, retrieved and flushed with 3000 ml physiological saline (Figure 1c). (4) After cylindrical reaming of the proximal femur, the prosthesis was inserted and cemented (Figure 1d). The long-stem femoral component was inserted into the inactivated bone off the table by carefully pressurizing cement into the inactivated bone, using the operator’s thumb to occlude the proximal medullar canal. Any excess cement was removed from the protruding stem of the femoral component and from the distal end of the inactivated bone. (5) After polymerization of the cement, the composite prosthesis was cemented into the host bone, and care was taken so no cement was caught between the inactivated autograft and the host bone. In all patients, autogenous iliac bone grafts were placed at the inactivated autograft-host bone junction to form extracortical grafting (Figure 1e).

**Figure 2:**
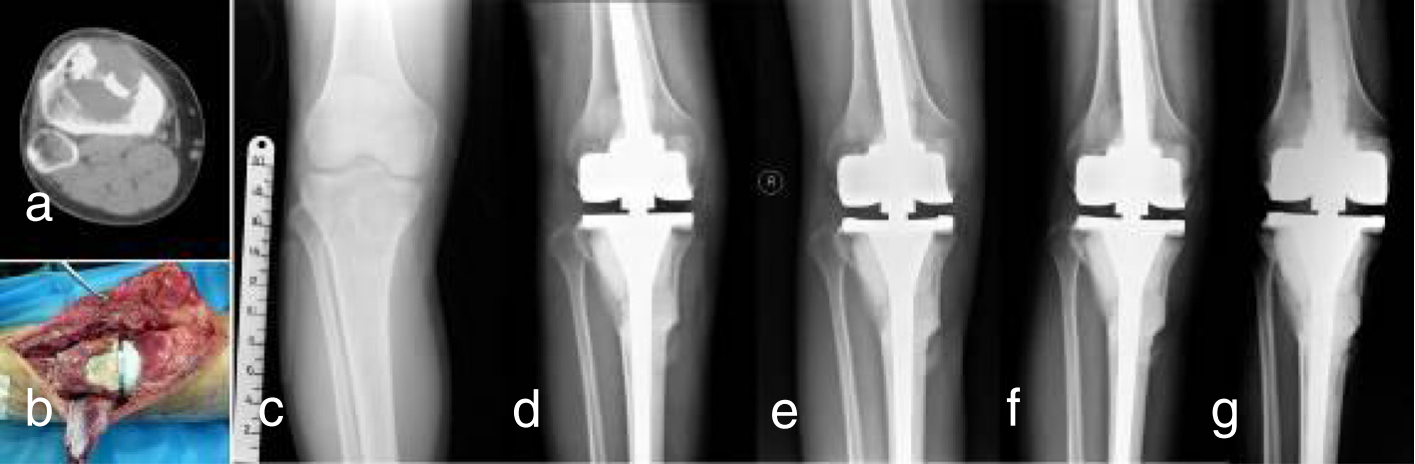
**A patient with postoperative recurrence of GCT in right proximal tibia (Case 6). a** Preoperative CT image showed the diameter of GCT lesion with soft tissue mass was over 1/2 on CT transversal image, **b** Extracortical grafting and medial gastrocnemius muscle flap transfer should be noted during operation, **c** Destruction of bone was shown on preoperative X-ray image, **d** After one week, good position of autograft-prosthesis composite was shown, **e** Bone callus was obvious 6 weeks after operation, **f** Osteotomy line dismissed and bone healing was found after 1 year. **g** After 3 years, the autograft-composite was in good position. MSTS score was 29 and ISOLS score was 35.

For GCT in proximal tibia, we think it was important to use the medial gastrocnemius muscle flap transfer to minimize the rate of complication, such as infection and skin necrosis (Figure 2b). The patellar tendon was sutured directly to the transposed flap to repair the extensor mechanism. The continuity of intact side of tumor bone with pathological fracture should be maintained during the inactivation procedure. The larger bone fragments should be cemented to inactivated tumor after alcohol inactivation.

Prophylactic antibiotics were administered for 48 hours. Low-molecular-weight heparin was administered for 2 weeks. For postoperative treatment, patients with lesion in distal femur were placed on bed for 2 weeks and in proximal tibia for 3 weeks. After that, partial weight bearing was allowed initially, and then weight bearing using two elbow crutches was allowed. Full weight bearing with no support was allowed at the end of 3 months. Plain anteroposterior and lateral radiographic examinations were done every 3 months for two years, bi-annually for a further three years and annually thereafter.

Limb function was evaluated with the Musculoskeletal Tumor Society (MSTS) rating scales, which comprise six items, namely pain, function, emotional acceptance, supports, walking and gait. The highest possible score is 30 and 5 points being allocated to each item [8]. Bone healing features were evaluated with the International Society of Limb Salvage (ISOLS) composite grafts evaluation method, which assesses bone remodeling, interface, anchorage, implant, fusion, resorption, fracture, graft shortening, fixation on plain X-ray radiographs and rates these items from 1 to 4 points [9].

## Results

All operations were performed successfully and the mean operating time was 2 hours and a half. No intraoperative complications occurred and the mean bleeding amount was about 450 ml.

The mean follow-up was for 54 months ranging from 38 to 47 months. 6 were followed for more than 4 years, and 2 were for more than 5 years. No recurrence, metastasis or composite removal occurred at the end of follow-up. No infection, composite fracture or prosthesis loosening was found. All patients were disease-free.

**Figure 3:**
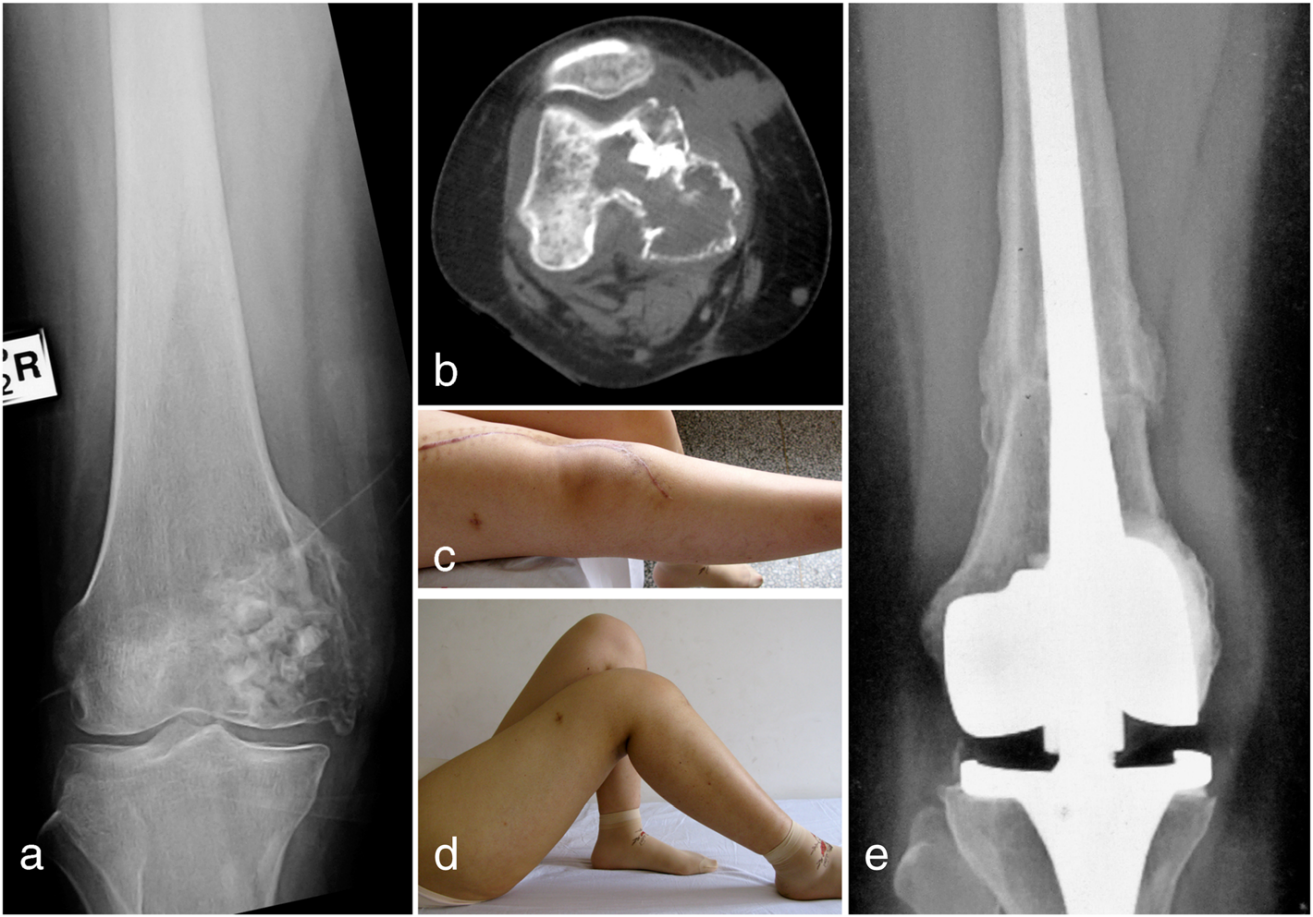
**A patient with postoperative recurrence of bone giant cell tumor in right distal femur was treated with wide resection of tumor and replacement of alcohol-deactived autograft-prothesis composite (Case 2). (a)** The preoperative X-ray image showed the osteolytic lesion encroaching articular surface and thinner cortical bone in medial condyle of right femur, **(b)** The preoperative CT image showed the lesion exceeding half of femoral diameter, **(c-d)** At 9-month after operation, the motion of right knee joint was 0°- 90°, **(e)** At 25-month after operation, the X-ray image showed bone healing and satisfactory space of prothesis without lossening and breakage, and the ISOLS graft score is 35.

The mean MSTS score was 26.3 (88%) ranging from 25 to 29 (Figure 3). The mean range of motion (ROM) in the knee joint was 100° ranging from 70° to 130°. The mean ISOLS composite graft score was 32.8 (88.5%) ranging from 28 to 35.3 in proximal tibia were over 30 (Figure 2). Bone union in composite-host bone junction site was found in all patients. The average union time was 5.5 months (range 4–7.5 months). No nonunion, loosening or resorption of host bone was found in all patients.

## Discussion

The ideal aim in the management of GCT is to eradicate the tumor and preserve joint function [10]. Wide resection is the treatment of choice, especially for GCT with extensive destruction of bone structure, recurrence, pathological fractures and difficulty in reconstruction after intralesional curettage [7,10]. Progress in biomedical engineering along with better surgical techniques has improved overall 10-year prosthetic survival rate after endoprosthetic replacement from 20% to 80% in the past three decades. As the survival of prosthesis improves, the major concern is its reconstruction longevity. However, it is difficult to overcome the mentioned disadvantages of prosthesis. It was believed by us that the alcohol inactivated autograft-prosthesis composite could be chosen for Campanacci grade III GCT of which the lesion diameter was over 1/2 on CT transversal image, with or without recurrence and pathological fracture.

The main aims of bone and joint reconstruction are: (1) skeletal stabilization, (2) restoration of length and alignment, and (3) preservation or reconstruction of optimum function [11]. It has been recommended that the most effective and easiest way to reconstruct the extensor mechanism sometimes around the knee is to use an allograft-prosthetic composite [12].

Achieving complete ablation of the tumor and preserving a functional extremity at the same time proves to be a difficult task due to the various anatomical factors unique to this site. Difficulties in the local control of giant cell tumors with Campanacci III as well as high rate of local recurrence following initial surgery have led the investigators to use different surgical modalities of reconstruction. At present, the most popular biological reconstruction method following skeletal tumor resection is allografting [13-16]. However, there are problems relating to infectious transmission, immunological reaction and refusal based on social or religious beliefs especially in Asian countries. Under these circumstances, recycled tumor bone autografts are widely used as an alternative to bone allografts. Devitalized bone autograft is particularly well suited in the region where allografts are not readily available [17]. Techniques that are capable of destroying tumor cells in resected bone include (1) irradiation [14,16], (2) autoclaving [18], (3) pasteurization [19,20], (4) freezing-thawing with liquid nitrogen [21] and alcohol inactivation [22,23]. Harrington et al. [18] reported that the autoclaving technique preserved a graft strong enough to support a joint arthroplasty and to allow early weight-bearing with a low risk of pseudarthrosis or late fatigue fracture. Jeon et al. [19] treated 15 distal femoral osteosarcoma patients who underwent reconstructions using pasteurized autograft–prosthesis composite. They found union of the osteotomy site was identified in ten patients (67%) of whom the average union time for junction sites was 16 months. Nonunion was identified in five patients, who had cemented fixation and a relatively long stem length in host bone. Loosening of the stem was found in three patients. Resorption of host bone was detected in two patients with noncemented fixation. Biau et al. [14] reported on irradiated allograft-prosthesis composites for proximal tibia reconstruction, it yielded poor results for proximal tibial reconstruction as complications and failures were common.

Compared to other methods, alcohol inactivation method is considered on the same level of safety in oncological control, which superiorities are economic-applicable to patients and the well fitness of bone graft with the defects. The rationale for this alcohol inactivated autograft-prosthesis composite combined the suitable bone and mega-prosthesis is the cumulative advantage provided by the biological properties of the former with the mechanical endurance of the latter. As shown in Table 2, there were no nonunion in our alcohol inactivated autograft group, better than that of allograft (25% [13], 12.9% [15]), irradiated allograft (73% [15], 50% [16]) and pasteurized autograft (33% [19]). No infection was found in our group, while 8.3-24.2% [13,15] in allograft. The MSTS score was 26.4 (88%) in our group which was near to that of allograft (90.4% [15]), better than that of pasteurized autograft (86% [19]) and irradiated allograft (67% [16]).

**Table 2:** **Literature review of autograft**/**allograft**-**prosthesis composite for neoplasms around knee**

**Study**	**Site**	**Number of patients**	**Average follow-up (months)**	**Type of tumor**	**Type of reconstruction**	**Reconstruction survival**	**MSTS functional score**	**Infection rate**	**Nonunion rate**
Current series	Around knee	8	54	GCT	Autograft-prosthesis composite	100%	88%	0	0
Song (2012) [20]	Proximal tibia	25	>10 years	Malignant tumors	Pasteurized autograft-prosthesis composite	68.7%	83.6%	20%	NA
Gilbert (2009) [13]	Proximal tibia	12	49	NA	Allograft-prosthesis composite	NA	81%	8.3%	25%
Donati (2008) [15]	Proximal tibia	62	72	56 malignant tumors	Allograft-prosthesis composite	73%	90.4%	24.2%	12.9%
				4 GCT					
				2 failure operation					
Jeon (2007) [19]	Distal femur	15	56	Osteosarcoma	Pasteurized autograft-prosthesis composite	100%	86%	0	33%
Biau (2007) [14]	Proximal tibia	26	128	NA	Irradiated allograft-prosthesis composite	79.4%	NA	23%	73%
Wunder (2001) [16]	Around knee	6	NA	Bone sarcoma	Irradiated allograft-prosthesis composite	50%	67%	18%	50%

The disadvantage of alcohol inactivated bone is that it needs a long time to accomplish revascularization and to integrate with surrounding bone. The rationality of alcohol inactivation is that alcohol could devitalize the tumor bone shell. The tumor cells had been devitalized when the ingrowth of surrounding vessels occurre [24]. Our previous studies showed that continuous bone callus presented after 8 weeks and complete bony healing showed after 12 weeks in rabbit femur [25]. The irradiated allograft presented the likely bony healing process of creeping substitution with the bone formation rate of 1 cm per 10 months [17]. In this study, according to the dynamic imaging observation and ISOLS composite scoring, the new bone originated from host bone and the bone healing time in femur is about 4 to 6 months and that in tibia about 6–8 months. We hold the viewpoint that creeping substitution is possibly the main way in bony junction and the healing time in femur is faster than that in tibia.

Metal prosthesis resulted in artifacts during CT and MRI imaging, which impaired the evaluation of bone healing. On this consideration, X-ray imaging was chosen to evaluate bone healing according to ISOLS composite grafts evaluation method [26]. The mean ISOLS composite graft score was 32.8 (88.5%) as good in our group ranging from 28 to 35 which were all over 28 as better. The average union time was 5.5 months (range 4–7.5 months) which was better than that of allograft (7.4 months) [27] and pasteurized autograft (16 months) [19]. The alcohol inactivated autograft was superior to allograft in bone healing. In addition, it was recommended by us that good anatomic reduction and specially made long stem play important roles in bone healing. If the lesion extent was over half of the affected bone and the host bone with enough strength, the patient should undergo *en bloc* resection of tumor and reconstruction with alcohol inactivated autograft–prosthesis composite.

There are limitations in this study, such as small amount of patients and the less than 5 years’ follow-up, which might result in biased result. The multi-centric randomized controlled study with large samples should be considered.

## Conclusions

Based on our outcomes, we believe alcohol inactivated autograft-prosthesis composite is a reasonable option as a limb salvage procedure for Grade III GCT around knee. By using this technique we have been able to achieve satisfactory oncological and functional outcomes in these patients.

## Consent

Written informed consent was obtained from the patient’s guardian/parent/next of kin for the publication of this report and any accompanying images.

## Competing interests

The authors declare that they have no competing interests.

## Authors’ contributions

XCY is leading the co-ordination of the study. SFX wrote this manuscript. All authors participated in the study design, provided feedback on drafts of this paper and read and approved the final manuscript.

## Pre-publication history

The pre-publication history for this paper can be accessed here:

http://www.biomedcentral.com/1471-2474/14/319/prepub
